# Macrophages in the synovial lining niche initiate neutrophil recruitment and articular inflammation

**DOI:** 10.1084/jem.20220595

**Published:** 2023-04-28

**Authors:** Kristina Zec, Barbora Schonfeldova, Zhichao Ai, Erinke Van Grinsven, Gabriela Pirgova, Hayley L. Eames, Dorothée L. Berthold, Moustafa Attar, Ewoud B. Compeer, Tal I. Arnon, Irina A. Udalova

**Affiliations:** 1 Kennedy Institute of Rheumatology, University of Oxford, Oxford, UK

## Abstract

The first immune-activating changes within joint resident cells that lead to pathogenic leukocyte recruitment during articular inflammation remain largely unknown. In this study, we employ state-of-the-art confocal microscopy and image analysis in a systemic, whole-organ, and quantitative way to present evidence that synovial inflammation begins with the activation of lining macrophages. We show that lining, but not sublining macrophages phagocytose immune complexes containing the model antigen. Using the antigen-induced arthritis (AIA) model, we demonstrate that on recognition of antigen–antibody complexes, lining macrophages undergo significant activation, which is dependent on interferon regulatory factor 5 (IRF5), and produce chemokines, most notably CXCL1. Consequently, at the onset of inflammation, neutrophils are preferentially recruited in the vicinity of antigen-laden macrophages in the synovial lining niche. As inflammation progresses, neutrophils disperse across the whole synovium and form swarms in synovial sublining during resolution. Our study alters the paradigm of lining macrophages as immunosuppressive cells to important instigators of synovial inflammation.

## Teaser

Using antigen-induced arthritis (AIA) model, Zec et al. show that synovial lining macrophages undergo IRF5-dependent activation and produce CXCL1 as a result of antigen recognition. Consequently, neutrophils are preferentially recruited in the synovial lining niche at the onset of inflammation.

## Introduction

Rheumatoid arthritis (RA) is a chronic autoimmune disease of the joints characterized by leukocyte recruitment into the synovium, the joint-supporting tissue, as well as the articular cavity (Smolen et al., 2018). Healthy synovium consists of the lining layer, which surrounds the joint cavity, and the sublining layer, which is positioned deeper in the tissue. Both lining and sublining are highly vascularized and can therefore support cell recruitment during inflammation, albeit the majority of synovial capillaries lie just below the lining (Knight and Levick, 1983).

Both the lining and sublining layers contain fibroblasts and resident macrophages scattered among adipocytes of the fat pad in the interstitium (Smith, 2011). Recent characterization of synovial tissue-resident macrophages in unprecedented detail (Alivernini et al., 2020; Culemann et al., 2019; Zhang et al., 2019) highlighted the fact that lining and sublining macrophages have substantially different phenotypes, being shaped by the microenvironment and their position. Nine macrophage populations have been identified in healthy controls, treatment-naive/-resistant RA patients, and the ones in sustained remission (Alivernini et al., 2020). However, it remains unclear how these distinct resident macrophages become activated in RA, i.e., what constitutes the break of tolerance in these cells. The expression of several immunosuppressive markers, such as VSIG4, TREM2, and MerTK (Alivernini et al., 2020; Culemann et al., 2019; Zhang et al., 2019) by lining macrophages and their depletion during active RA has led to the assumption that lining macrophages negatively regulate joint inflammation. Nevertheless, this hypothesis has never been tested directly within intact joint tissue at the onset of inflammation.

Lining macrophages have direct access to the synovial cavity, and one of the first immune-activating events in mouse arthritis models involves the formation of immune complexes (ICs) on the surface of the synovial cavity (Miyabe et al., 2017). Given that lining macrophages phagocytose autoantibodies (Culemann et al., 2019), we hypothesized that synovial inflammation begins with the activation of these cells, leading to the recruitment of neutrophils. In this study, we have utilized a spatiotemporal imaging approach in a systematic, whole-organ, and quantitative way to dissect the first cellular and molecular events at the onset of joint inflammation. Firstly, we show that lining macrophages, which can be identified by the expression of the surface protein, VSIG4, as well as high level of CX3CR1 expression, preferentially phagocytose ICs containing the model antigen. Using the antigen-induced arthritis (AIA) model of articular inflammation, we confirm that lining macrophages phagocytose methylated BSA (mBSA) containing ICs and become activated, leading to the production of neutrophil recruiting chemokines, such as CXCL1. Neutrophils are the most abundant cell type recruited into the joint during inflammation in murine models of RA, and they have also been implicated in driving human disease (Velvart et al., 1981; Kundu et al., 2012). Here we show, for the first time, that the initial recruitment of neutrophils occurred in the vicinity of the synovial lining, where vascular endothelium expressed the activation marker E-selectin. We confirm that this process is reliant on the CXCL1/CXCR2 axis. Lining macrophage–targeted deletion of IRF5, a known driver of inflammatory macrophage phenotype (Krausgruber et al., 2011; Weiss et al., 2015), specifically impaired the activation of lining macrophages and their production of CXCL1 and reduced neutrophil recruitment into the synovial lining of the joint at the onset of AIA. Our study highlights lining macrophages as critical upstream instigators of joint inflammation and warrants further investigation into how the cell crosstalk in the lining can be targeted to abridge inflammation.

## Results and discussion

### Lining macrophages phagocytose antigen at the onset of articular inflammation

The previous studies of serum transfer–induced arthritis model of articular inflammation indicated that lining macrophages take up labeled IgG antibodies (Culemann et al., 2019). In the AIA model, animals are immunized with mBSA supported by complete Freund’s adjuvant and challenged by direct injection of mBSA into the synovial cavity (Brackertz et al., 1977). The resulting articular inflammation likely involves IC formation (Di Ceglie et al., 2018). However, it is unknown whether lining macrophages preferentially uptake this model antigen within the ICs.

**Figure 1.**
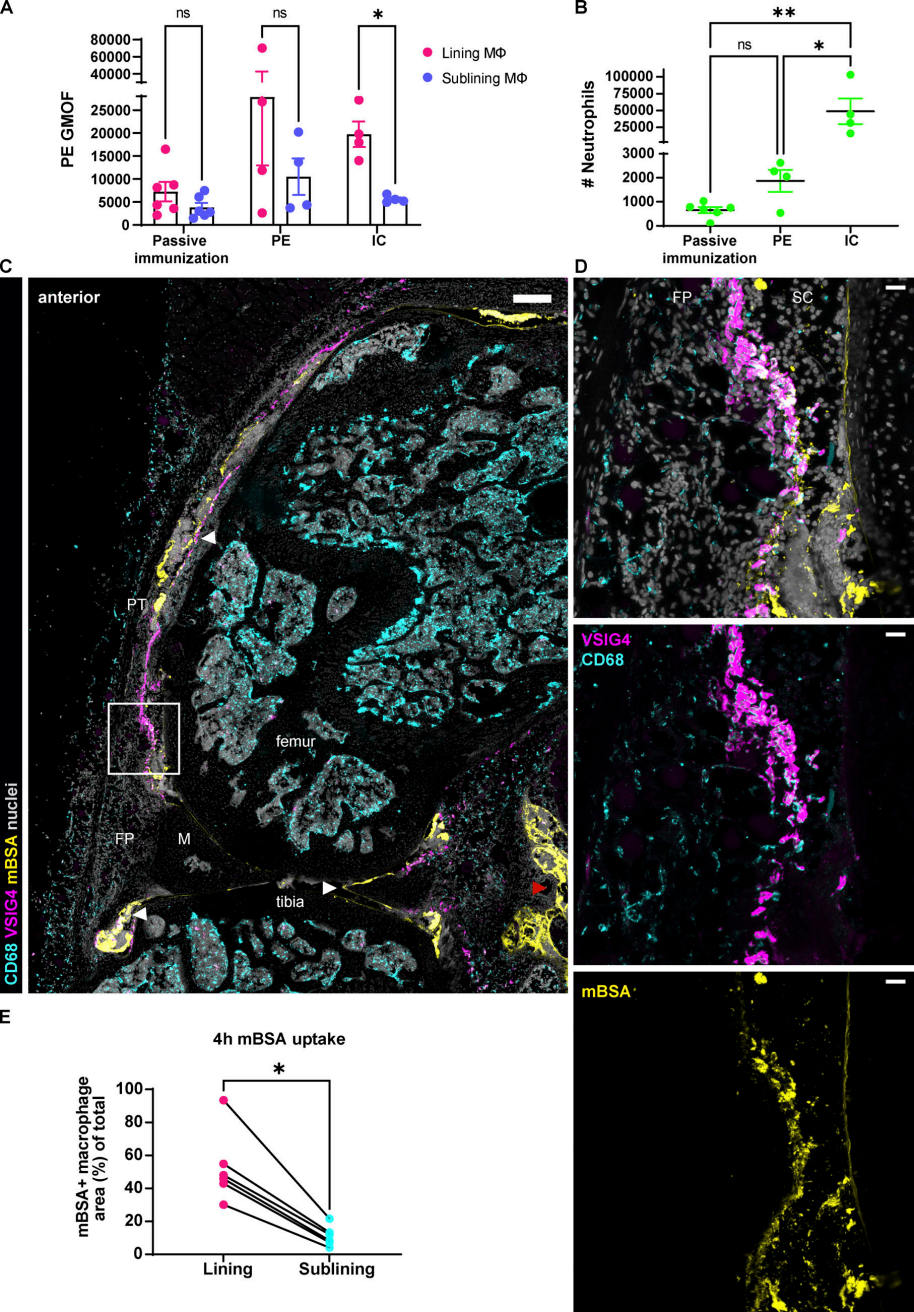
**Lining macrophages phagocytose ICs and model antigen-mBSA. (A)** Quantification of PE fluorescence intensity by FACS in synovial resident macrophages 3 h after IA injections. Significance values by mixed-effects analysis with Šídák multiple comparisons post hoc: *, P < 0.05. GMOF, geometric mean of fluorescence. **(B)** Synovial neutrophil counts as a result of IC and PE challenge by FACS at 3 h after IA injections. Significance values by one-way ANOVA with Šídák multiple comparisons post hoc: *, P < 0.05; **, P < 0.01; data representative of two experiments. **(C)** Confocal fluorescence microscopy of the whole mouse knee in sagittal plane 4 h after injection of fluorescently labeled mBSA (large panel with white arrowheads pointing out lining and cartilage localization of the antigen, whereas red arrowhead indicates leak in the posterior part; pan-macrophage CD68, cyan; lining macrophage VSIG4, magenta; mBSA, yellow; nuclei, gray; colocalization of VSIG4 and mBSA, white; scale bar = 100 µm). **(D)** Zoomed region of the lining shows preferential uptake of mBSA by the lining macrophages and not the sublining ones (three right panels, scale bar = 20 µm). **(E)** Quantification of mBSA uptake by the lining and sublining macrophages (*N* = 2; *n* = 6) in a scatter plot; each symbol indicates one section, and lines connect macrophages of the same section. *N* indicates the number of mice, *n* the number of quantified sections; data representative of two independent experiments. Significance values by Wilcoxon matched-pairs signed rank test: *, P < 0.05. FP, fat pad; M, meniscus; PT, patellar tendon; SC, synovial cavity.

**Figure S1.**
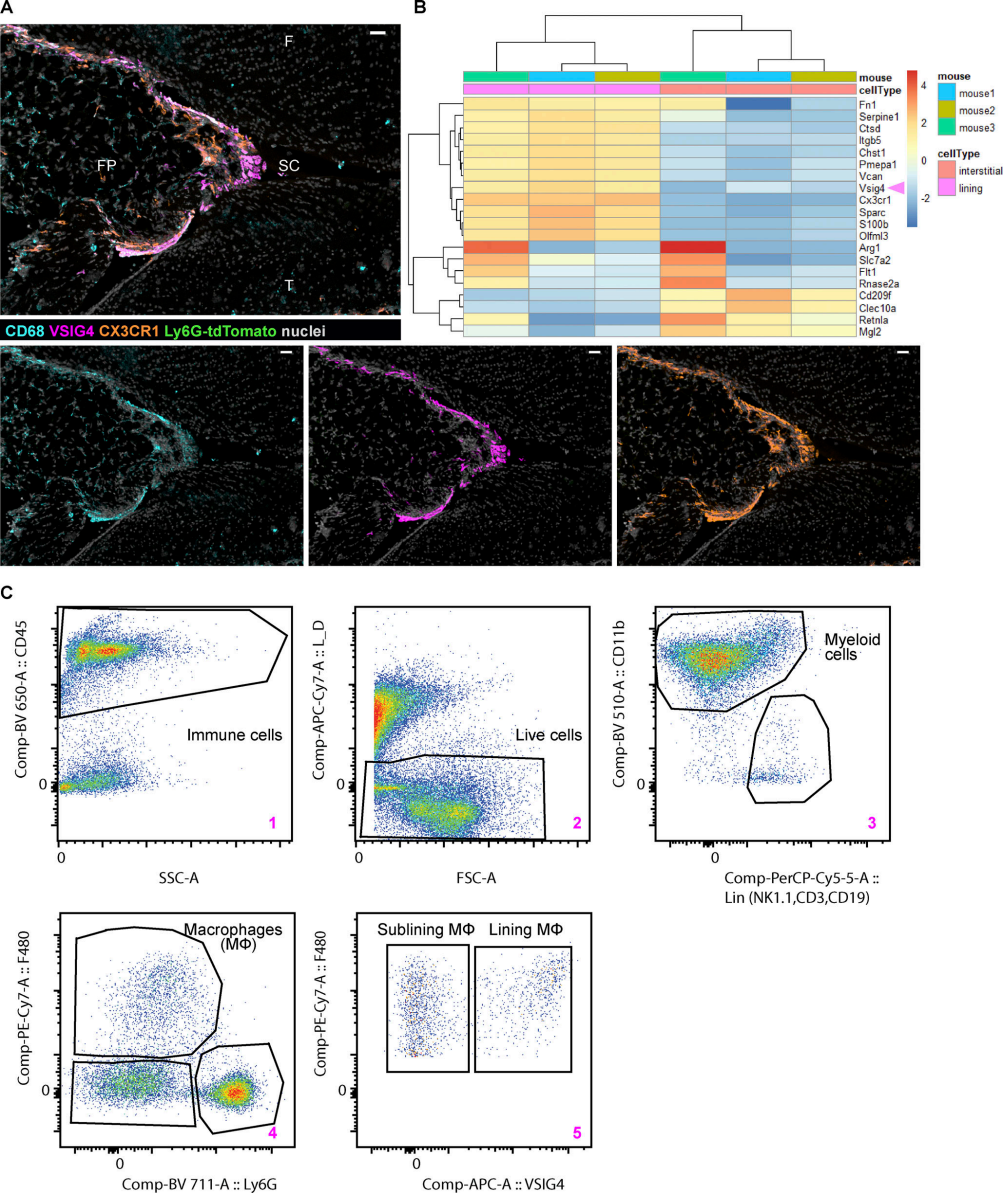
**Synovial lining macrophages can be identified and isolated by the expression of VSIG4. (A)** Confocal image of the naive knee from CX3CR1-eGFP Ly6G-tdTomato double reporter mouse containing resident macrophages and no neutrophils; VISG4 and CX3CR1 colocalize in the lining (pan-macrophage CD68, cyan; lining macrophage VSIG4, magenta; CX3CR1, orange; nuclei, gray; sagittal plane; scale bar = 30 µm). **(B)** VISG4 marks lining macrophages by reanalysis of publicly available small bulk RNA sequencing dataset of murine paws in steady state (Culemann et al., 2019). **(C)** Gating strategy used to isolate lining and sublining macrophages by FACS for small bulk RNA sequencing; pseudo-color plots of contralateral knee at 4 h p.c. created in FlowJo v10. F, femur; FP, fat pad; SC, synovial cavity; T, tibia.

We first compared a general capacity of lining and sublining macrophages to ingest a model antigen alone or in ICs. We have delineated lining macrophages in situ by the expression of VSIG4 (Fig. 1 and Fig. S1, A and B), a molecule also identified in other mouse (Culemann et al., 2019) and human studies (Zhang et al., 2019), as lining-specific even upon inflammation. We then used fluorochrome PE alone or in ICs (Fig. 1 A). We have either (1) passively immunized mice against PE by injecting anti-PE immunoglobulins i.v., followed by intra-articular (IA) PE administration, (2) injected PE alone into the knee, or (3) challenged the knee with the preformed PE ICs. PE fluorescence in macrophages and resulting neutrophil recruitment were analyzed 3 h after injection by FACS of the cells of disaggregated synovia. Lining macrophages ingested greater amount of preformed PE ICs compared to sublining macrophages (Fig. 1 A). Importantly, this was followed by the greatest neutrophil recruitment (Fig. 1 B). Passive immunization and PE alone did not lead to significant differences in ingestion between lining and sublining macrophages (Fig. 1 A) or neutrophil recruitment (Fig. 1 B).

We next visualized the spatial and cellular distribution of mBSA at the onset of inflammation in the AIA model. For this, we have fluorescently labeled this protein and performed confocal microscopy of the whole mouse knee at 4 h post challenge (p.c.; Fig. 1). We observed that mBSA formed extensive aggregates that were captured by the synovial lining or remained sticking to the cartilage (Fig. 1 C, large overview, indicated with white arrowheads). In the posterior part of the knee, mBSA also leaked out of the synovium into the muscle (Fig. 1 C, large overview, red arrowhead), perhaps unsurprising, as the injection is performed with the mouse in a supine position. Zooming in on the anterior part of the knee, we found that VSIG4^+^ lining macrophages (Fig. 1) phagocytosed a significant amount of mBSA (Fig. 1, D and E). In contrast, the sublining macrophages, marked as positive for the pan-macrophage marker CD68 but negative for VSIG4, took up substantially less mBSA (Fig. 1, D and E), similar to the observed phagocytosis of PE-containing ICs (Fig. 1 A).

Thus, lining macrophage activation depends on the uptake of antigen–antibody complexes, and these cells play a key role in the initial response of the synovium to an insult.

### Activated lining macrophages are potent producers of chemokines

**Figure S2.**
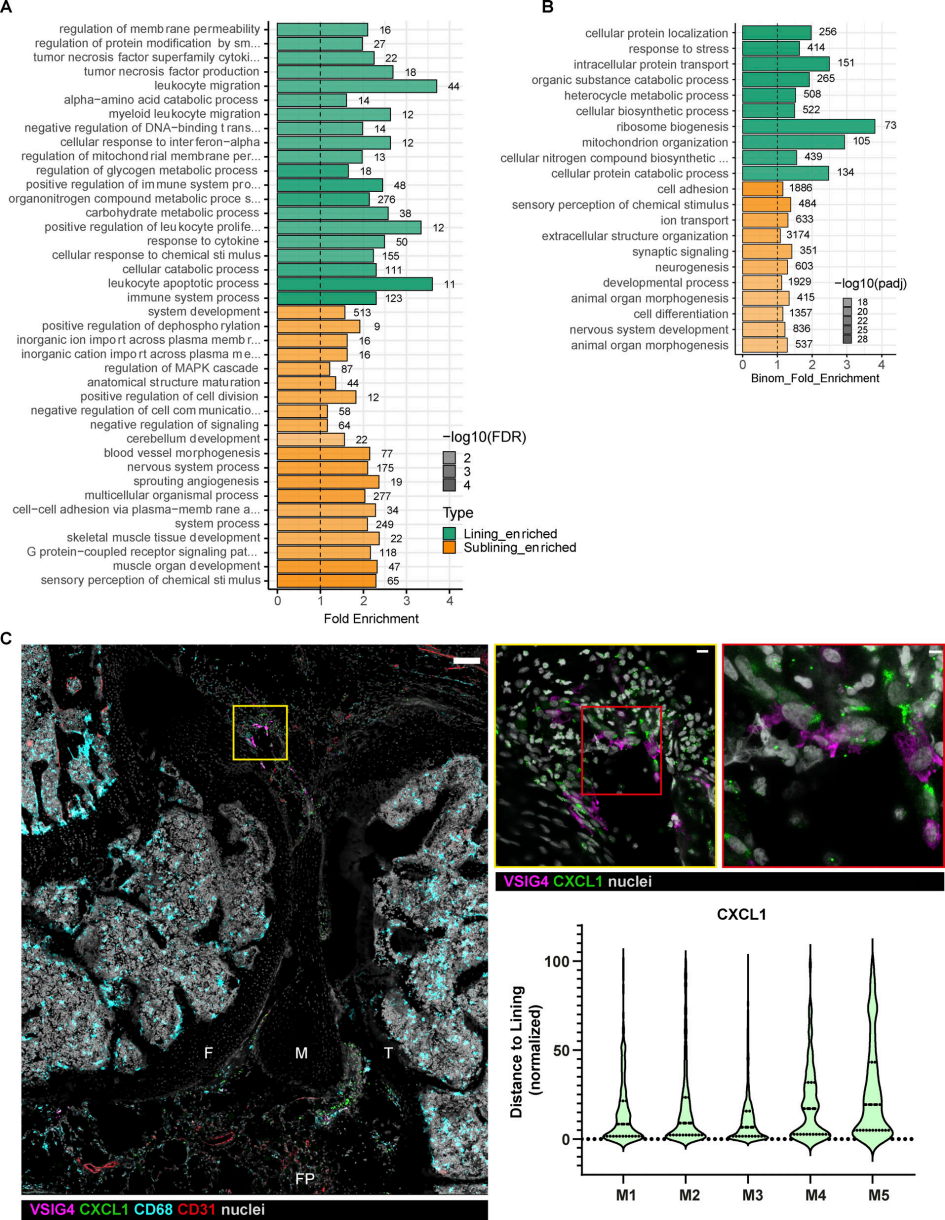
**Activated transcriptional profile in lining macrophages at the onset of AIA and CXCL1 expression in the lining. (A)** GO analysis of differentially expressed genes in lining and sublining macrophages at 4 h after mBSA challenge (*n* = 3). FDR, false discovery rate. **(B)** GO analysis of differentially expressed genes in lining and sublining macrophages from PBS control knees (*n* = 3). **(C)** Confocal images of the knee immunostained for CXCL1 at 4 h p.c. (large panel; VSIG4, magenta; CXCL1, green; CD68, cyan; CD31, red; nuclei, gray; scale bar = 100 µm). Zoomed region of the lining shows expression of CXCL1 in various cells (middle panel, scale bar = 10 µm), whereas further enlargement demonstrates colocalization of CXCL1 and VSIG4 (right panel, scale bar = 3 µm). **(D)** Quantification of CXCL1 distribution in the synovium in additional mice from two independent experiments; Each violin plot represents one to two knee sections of a single mouse with median and interquartile range indicated. F, femur; FP, fat pad; M, meniscus; T, tibia.

**Figure 2.**
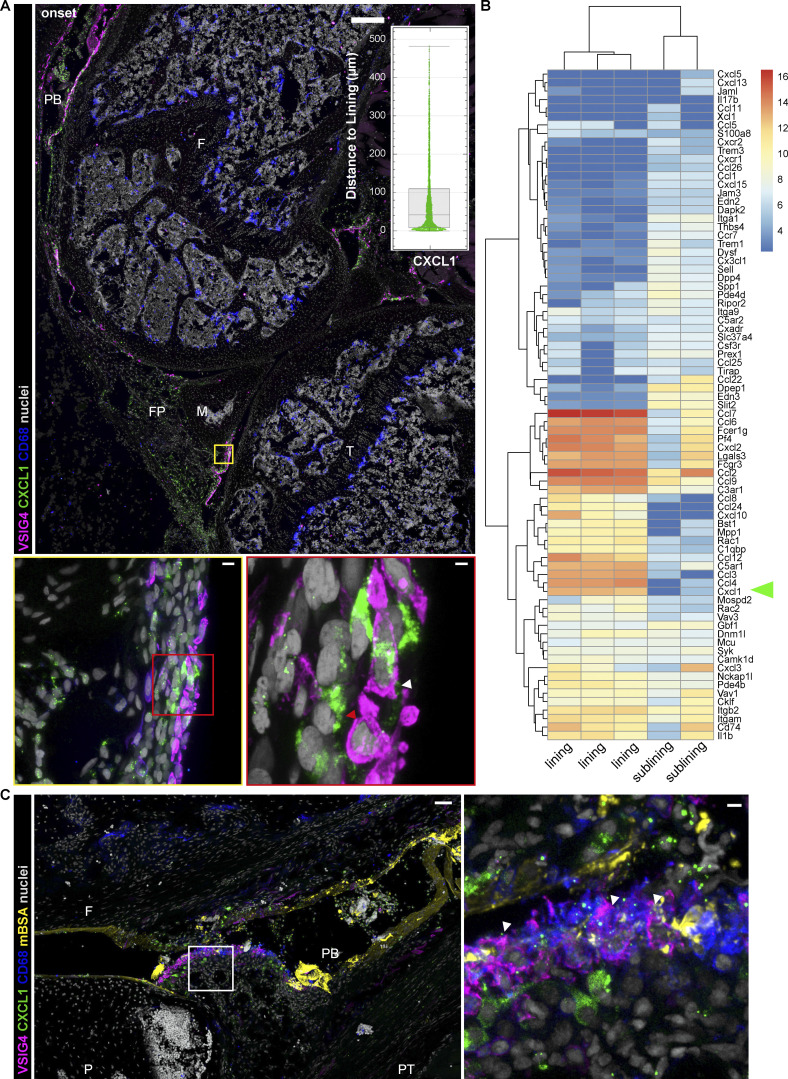
**Activation of lining macrophages at the onset of AIA. (A)** Confocal fluorescence microscopy of mouse knee demonstrates CXCL1 immunostaining localizing in the lining at 4 h after AIA induction (large panel; VSIG4, magenta; CXCL1, green; CD68, blue; nuclei, gray; scale bar = 200 µm) with representative quantification of CXCL1 expression in regards to the synovial lining of the image in A; each point is one CXCL1 “surface” rendered by Imaris software with median and interquartile range indicated. Zoomed region of the lining shows expression of CXCL1 in the cells of the lining niche (bottom left panel, scale bar = 7 µm), whereas further enlargement demonstrates colocalization of CXCL1 and VSIG4 indicated with a white arrowhead, as well as CXCL1 expression in VSIG4^−^ lining cell indicated with a red arrowhead (bottom right panel, scale bar = 2 µm). F, femur; FP, fat pad; M, meniscus; PB, patellar bursa; T, tibia. Images representative of 10 sections from five different mice and two independent experiments. **(B)** Heatmap of genes within the GO term “Neutrophil recruitment” expressed in synovial macrophages shows higher expression of chemokines in lining cells; CXCL1 indicated with an arrowhead. **(C)** Confocal image of sub-patellar synovial region demonstrating fluorescent mBSA and CXCL1 coincidence in the lining area (left panel; VSIG4, magenta; CXCL1, green; CD68, blue; mBSA, yellow; nuclei, gray; scale bar = 40 µm). Enlarged region shows lining macrophages with intracellular mBSA and CXCL1 expression (right panel, arrowheads, scale bar = 7 µm). F, femur; P, patella; PB, patellar bursa; PT, patellar tendon.

To understand the consequences of mBSA recognition by synovial macrophages at the onset of AIA, we have isolated the VSIG4^+^ F4/80^+^ lining and VSIG4^−^ F4/80^+^ sublining macrophages from immunized and challenged knees (Fig. S1, A–C) and performed small bulk RNA sequencing analysis (Fig. S2, A and B). Comparison between the lining and sublining macrophages in the inflamed knee revealed 4,873 differentially expressed genes (p-adj < 0.05), 2,587 of which are significantly upregulated by lining macrophages. Gene ontology (GO) analysis showed enrichment in immune system–related processes in the lining cells, e.g., “positive regulation of immune system process” and “response to cytokine,” indicating their more activated status, whereas processes related to organ development were enriched in sublining macrophages (Fig. S2 A). Further, lining macrophages upregulated genes within “tumor necrosis factor production” and “myeloid leukocyte migration” molecular pathways at the onset of AIA (Fig. S2 A). Genes within the GO term “neutrophil recruitment,” including several CCL (*CCL2*, *3*, *4*, *6*, *7*, *8*, *9*) and CXCL (*CXCL1*, *2*, *3*, *10*) chemokines, were more enriched in the lining compared with sublining macrophages (Fig. 2 B), suggesting their role in leukocyte recruitment upon exposure to the antigen.

The GO ontologies related to the immune system processes were not detected in the analysis of DE genes between lining vs. sublining macrophages from PBS control knees, which were largely limited to metabolic and bioenergetic categories (Fig. S2 B), suggesting that the lining macrophages get activated and assume their inflammatory and immune function at the onset of AIA.

As CXCL1 plays an important role in the pathogenesis of human RA (Koch et al., 1995; Hou et al., 2020), we embarked on validation of CXCL1 protein levels in situ. Indeed, confocal microscopy of the whole mouse knee revealed distinct localization of CXCL1 in the lining layer at 4 h p.c. (Fig. 2 A), an observation quantified by measuring the distance of CXCL1 signal from the lining layer (Fig. 2 A). This distinct lining localization was conserved across the whole knee and in different mice (Fig. S2 C). Employing high-resolution imaging, we identified that lining macrophages contain CXCL1 within their cell bodies (Fig. 2 A, bottom right panel, white arrowhead). However, close inspection also revealed that other cells in the lining layer produced CXCL1 as well (Fig. 2 A, bottom left panel and bottom right panel, red arrowhead). Further, topography of the CXCL1 protein on the synovial tissue sections resembles that of labeled mBSA, and it appears that the amount of CXCL1 contained in lining macrophages correlates with ingestion of labeled mBSA (Fig. 2 C). Given the preferential uptake of this antigen by lining macrophages (Fig. 1) and their activated transcriptional signature (Fig. 2 B and Fig. S2 A), we hypothesize that lining macrophages induce expression of CXCL1 in adjacent cells of the lining area, possibly through the secretion of an upstream mediator of inflammation, the exact nature of which remains to be determined.

### Neutrophils cluster in the lining at the onset of AIA

**Figure 3.**
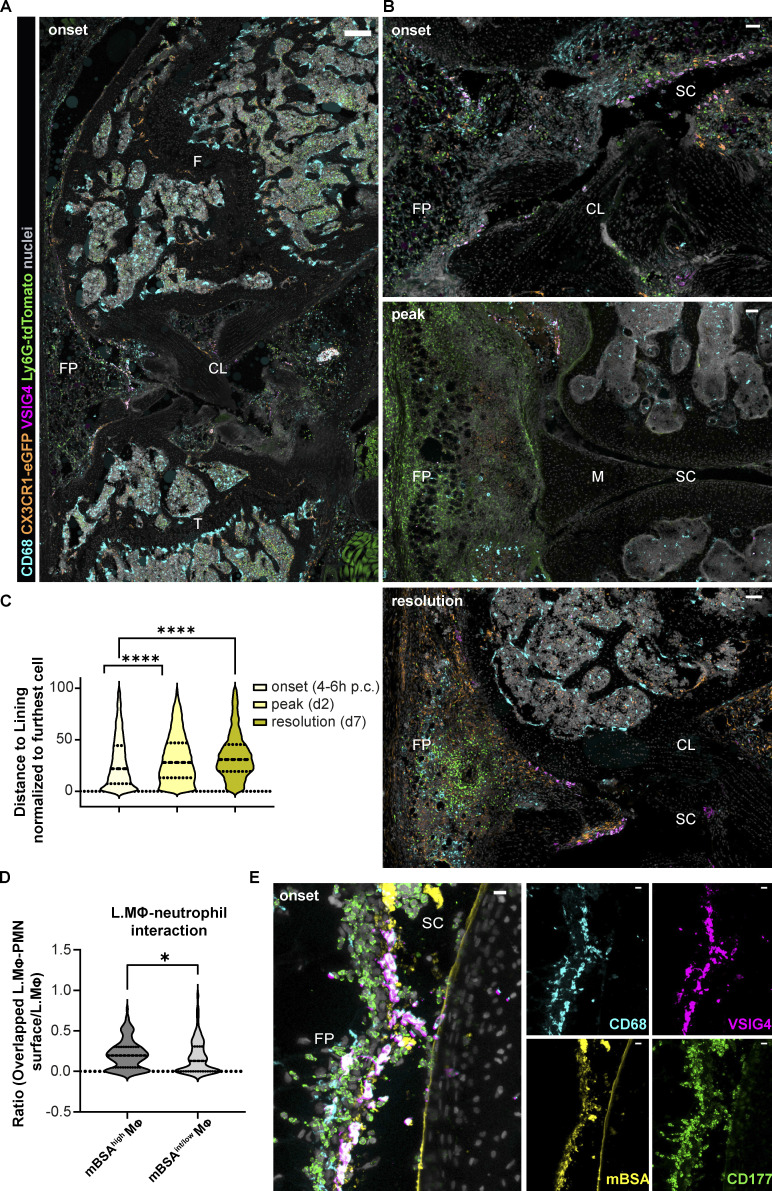
**Topography of recruited neutrophils during AIA: Onset-specific clustering in the synovial lining and interaction with mBSA-laden lining macrophages. (A and B)** Confocal images of murine synovia during AIA in CX3CR1-eGFP Ly6G-tdTomato double reporter mice show that neutrophils cluster in the synovial lining niche at the onset: 6 h large overview (A) and 4 h anterior synovium ROI (B) have uniform distribution at day 2 (peak of inflammation, middle panel) and form localized swarms at day 7 (resolution of inflammation, bottom panel). CD68, cyan; CX3CR1, orange; VSIG4, magenta; Ly6G-tdTomato, green; nuclei, gray; scale bar = 300 and 50 µm in the entire mouse knee cross-section and ROIs, respectively. **(C)** Respective quantification of neutrophil position within the anterior synovium of the whole knee cross-sections as in A: neutrophil distance to the lining at the onset (6 h), peak (day 2), and resolution (day 7) of AIA presented in violin plots. Each violin plot represents three mice combined with two to three sections at different positions in the knee quantified, median and interquartile range indicated. Total quantifications: *N* = 3; *n* = 2–3; *N* indicates mouse group size, *n* the number of sections per mouse; samples from four independent experiments. P values by Kolmogorov–Smirnov comparison: ****, P < 0.0001. CL, cruciate ligaments; F, femur; FP, fat pad; M, meniscus; SC, synovial cavity. **(D)** Lining macrophages (L.MΦ) exhibiting higher mBSA uptake interact with more neutrophils (PMN) compared with the macrophages with lower mBSA uptake (whole anterior lining quantified on two different sections of a single mouse at 4 h p.c.; P value by Wilcoxon rank–sum test: *, P < 0.05). **(E)** Confocal image of the anterior synovium 4 h after injection of fluorescently labeled mBSA shows recruited neutrophils interacting with mBSA-laden lining macrophages (CD68, cyan; VSIG4, magenta; mBSA, yellow; neutrophil CD177, green; colocalization of VSIG4 and mBSA, white; scale bar = 10 µm). FP, fat pad; SC, synovial cavity.

**Figure 4.**
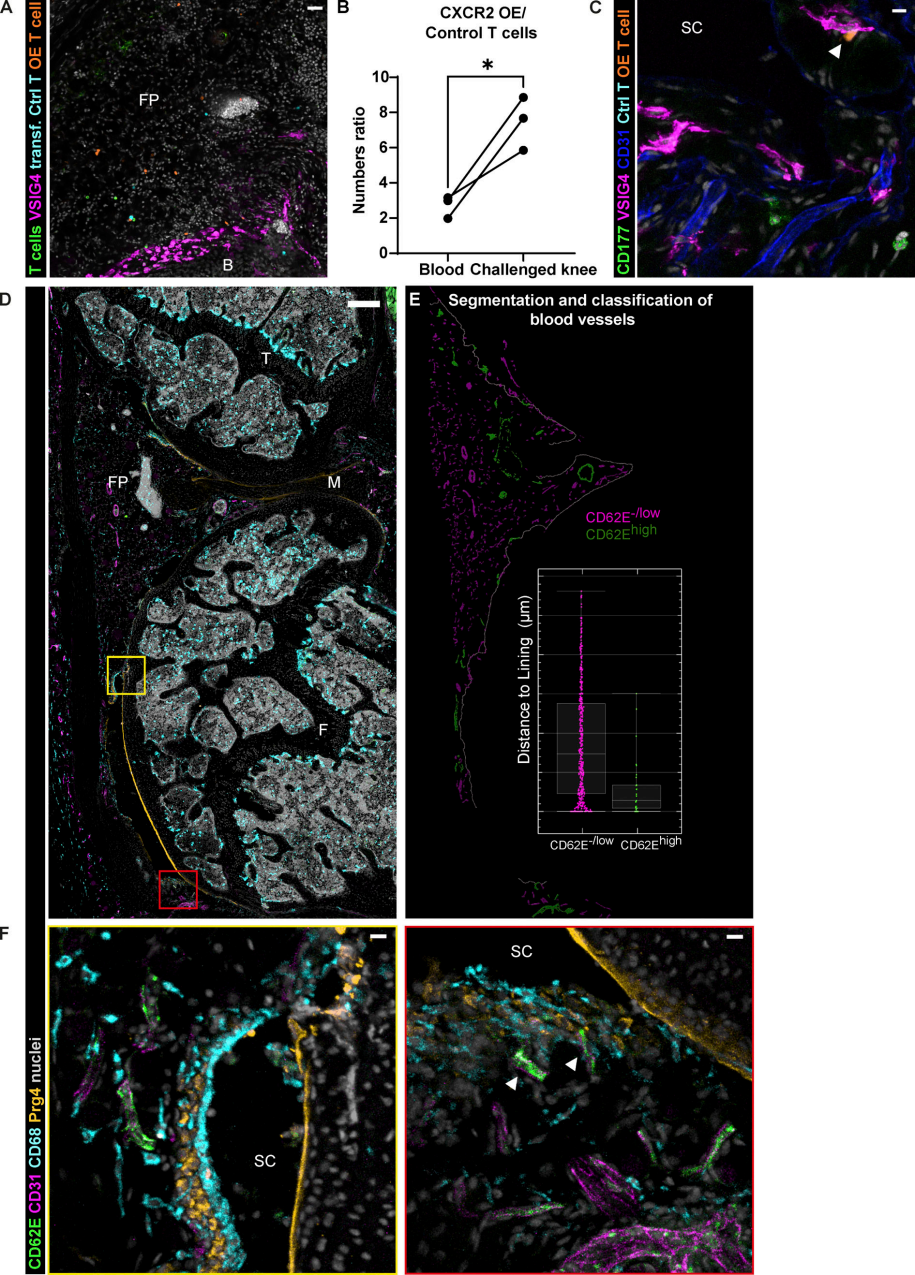
**Activation of microvasculature in the synovial lining niche at the onset of AIA. (A)** Representative confocal image of the synovium demonstrating higher number of transferred CXCR2 OE T cells compared with the control T cells and the closer proximity of OE T cells to the lining (CD4^+^; CD8^+^ T cells, green; VSIG4, magenta; control T cells, cyan; CXCR2 OE T cells, orange; nuclei, gray; scale bar = 30 µm); single experiment with two animals. **(B)** Numbers ratio of CXCR2 OE T cells to control T cells measured by FACS in the blood and the synovium of mice 6 h p.c. (5 h after adoptive T cell transfer) shows preferential recruitment of CXCR2 OE T cells to the synovium. Significance value by paired *t* test: *, P < 0.05. Experiment performed two times. **(C)** CXCR2 OE T cells in the synovial cavity interacting with the lining macrophage (CD177, green; VSIG4, magenta; CD31, blue; control T cells, cyan; CXCR2 OE T cells, orange; nuclei, gray; scale bar = 10 µm). **(D)** Confocal image of the whole mouse knee cross-section immunostained for endothelial activation marker E-selectin (CD62E) reveals distinct position of activated vasculature close to the lining (CD62E, green; CD31, magenta; CD68, cyan; lining fibroblast Prg4, orange; nuclei, gray; scale bar = 200 µm). **(E)** Segmentation of the vasculature in the anterior synovium of the image in A using Imaris to classify highly activated (CD62E^high^) and non-activated (CD62E^−/low^) blood vessels. Inset shows representative distribution of activated microvasculature in regards to the synovial lining of the image mask in E; each point is one blood vessel surface of the respective image; median and interquartile range indicated. **(F)** Enlarged region of the synovial lining with CD62E^high^ blood vessel is shown in the left panel, whereas the right panel shows patellar region with CD62E^high^ blood vessels in direct contact with lining macrophages (arrowheads), as well as an activated larger-diameter blood vessel situated a few cell layers deeper (scale bar = 20 µm). B, bone; F, femur; FP, fat pad; M, meniscus; SC, synovial cavity; T, tibia. Images and quantification representative of four animals from two independent experiments.

Neutrophil entry site into the synovium during articular inflammation has not been defined so far, and the role of lining vs. sublining macrophage subsets in mediating this process has not been determined. We thus examined the pattern of neutrophil recruitment into the synovium at the onset (4–6 h), peak (day 2), and the beginning of resolution of inflammation (day 7; Fig. 3). Segmentation of neutrophils identified using the Ly6G-tdTomato reporter mice and/or CD177 immunostaining was carried out on the entire anterior part (fat pad and patellar region) of whole knee cross-sections, as shown in the image representing 6 h after mBSA challenge (Fig. 3 A), and their distance from the lining was measured and normalized to the furthest cells for intra- and intersample comparison (Fig. 3 C). As expected, neutrophils were absent in naive synovia (Fig. S1 A). Both visual inspection (Fig. 3, A and B) and quantification (Fig. 3 C) of neutrophil patterns confirmed that they clustered close to the lining at the onset of inflammation (Fig. 3 C). Moreover, we observed that neutrophils surrounded and interacted specifically with mBSA-laden lining macrophages (Fig. 3, D and E), emphasizing the role of lining macrophages in this process. Moreover, the amount of ingested mBSA by lining macrophages correlated with the number of neutrophils in direct contact with these cells (Fig. 3 D). Importantly, the clustering phenomenon was lost at the peak of inflammation with neutrophils being ubiquitously distributed in the tissue (Fig. 3 B, middle panel), as well as during the resolution when they formed localized swarms (Fig. 3 B, bottom panel). To demonstrate the causality between CXCL1 expression in the lining niche and local cell recruitment, we have adoptively transferred a 1:1 mix of syngeneic control and CXCR2 overexpressing (OE) T cells 1 h after IA mBSA injection (Fig. 4, A and B). We have observed more CXCR2 OE T cells compared with the control T cells in both blood and the synovium, expressed as the ratio of OE to control T cells in both compartments. However, the ratio of CXCR2 OE T cells to the control ones was significantly greater in the synovium compared with the blood (Fig. 4 B). Importantly, the CXCR2 OE T cells localized close to the lining macrophages (Fig. 4 A) and occasionally formed direct contact with them (Fig. 4 C, arrowhead), a phenomenon we couldn’t observe for control T cells, which in turn mostly accumulated in the bone marrow (data not shown). Taken together, these data support the hypothesis that joint inflammation begins in the synovial lining with the activation of lining macrophages, local production of CXCL1, and recruitment of the first wave of neutrophils into the lining niche.

### Activated endothelium in the lining may support localized neutrophil recruitment

**Figure S3.**
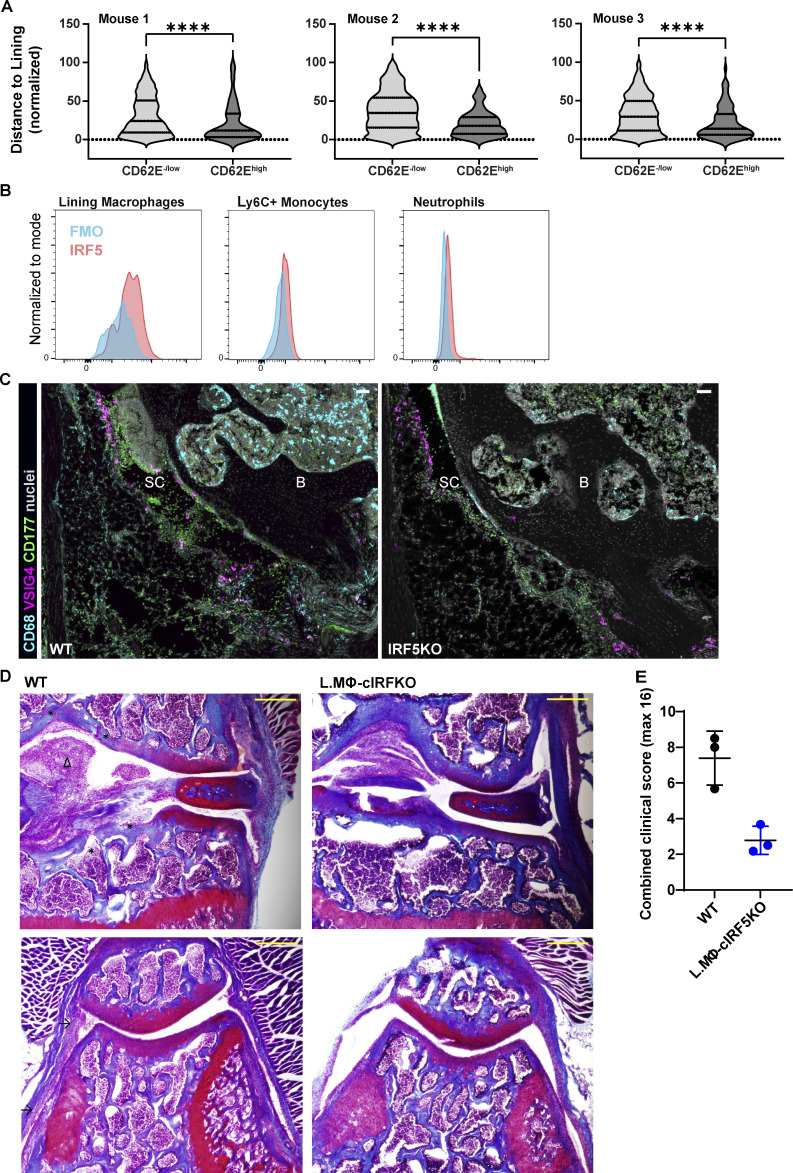
**Visualizing activated endothelium and IRF5-dependent activation of the synovial lining. (A)** Distance of activated and non-activated vasculature to the lining at 4 h p.c. in three different mice from two experiments. Each violin plot represents two to three knee sections of a single mouse with median and interquartile range indicated. P values by Wilcoxon rank–sum test: ****, P < 0.0001. **(B)** Staining of IRF5 demonstrates expression in VISG4^+^ lining macrophages that is higher compared to inflammatory monocytes and neutrophils by FACS. **(C)** Representative confocal images of the synovia from WT and IRF5KO mice show fewer neutrophils in IRF5KO mice, in line with FACS data (CD68, cyan; VSIG4, magenta; CD177, green; nuclei, gray; scale bar = 50 µm). B, bone; SC, synovial cavity. **(D)** Representative images of Fast Green and Safranin O–stained knee sections from a single experiment in WT and CX3CR^CreER^ IRF5^fl/fl^ at day 7 p.c. (upper panels, articular surface region; lower panels, patellar region; asterisks indicate bone erosion, arrows synovial hyperplasia, and arrowheads fat pad infiltration; coronal orientation; scale bar = 300 µm). **(E)** Clinical scoring of respective sections for synovial hyperplasia, bone erosion, and BM proliferation shows improved pathology in lining macrophage–specific IRF5KO mice.

We next investigated whether neutrophil recruitment to the lining layer is supported by enhanced extravasation at specific sites of the vasculature. To examine the topography of activated blood vessels in the synovium, we have performed immunostaining for activated endothelium using a combination of CD31 and E-selectin (Fig. 4). E-selectin is robustly and transiently expressed on endothelial cells in response to inflammatory mediators such as TNFα (Wyble et al., 1997) and IL-1β (Montgomery et al., 1991). Moreover, E-selectin blockade with antibodies has been shown to decrease neutrophil recruitment in adjuvant-induced arthritis in rats (Issekutz et al., 2001). Analogous to CXCL1 and neutrophil localization, blood vessels expressing E-selectin predominantly lied close to the synovial lining (Fig. 4, D and E; and Fig. S3 A). Noteworthy, lining macrophages directly interacted with the vasculature in many instances (Fig. 4 F, right panel, marked with arrowheads). We also observed activated blood vessels deeper in the synovium, surrounded by the sublining macrophages, which could be relaying the stimulating signal from the lining (Fig. 4 F, right panel). Thus, the predominance of E-selectin–positive blood vessels in the lining vicinity corroborates the notion of lining activation at the onset of joint inflammation, leading to the privileged neutrophil extravasation in the area.

### IRF5 orchestrates activation of lining macrophages at the onset of AIA

**Figure 5.**
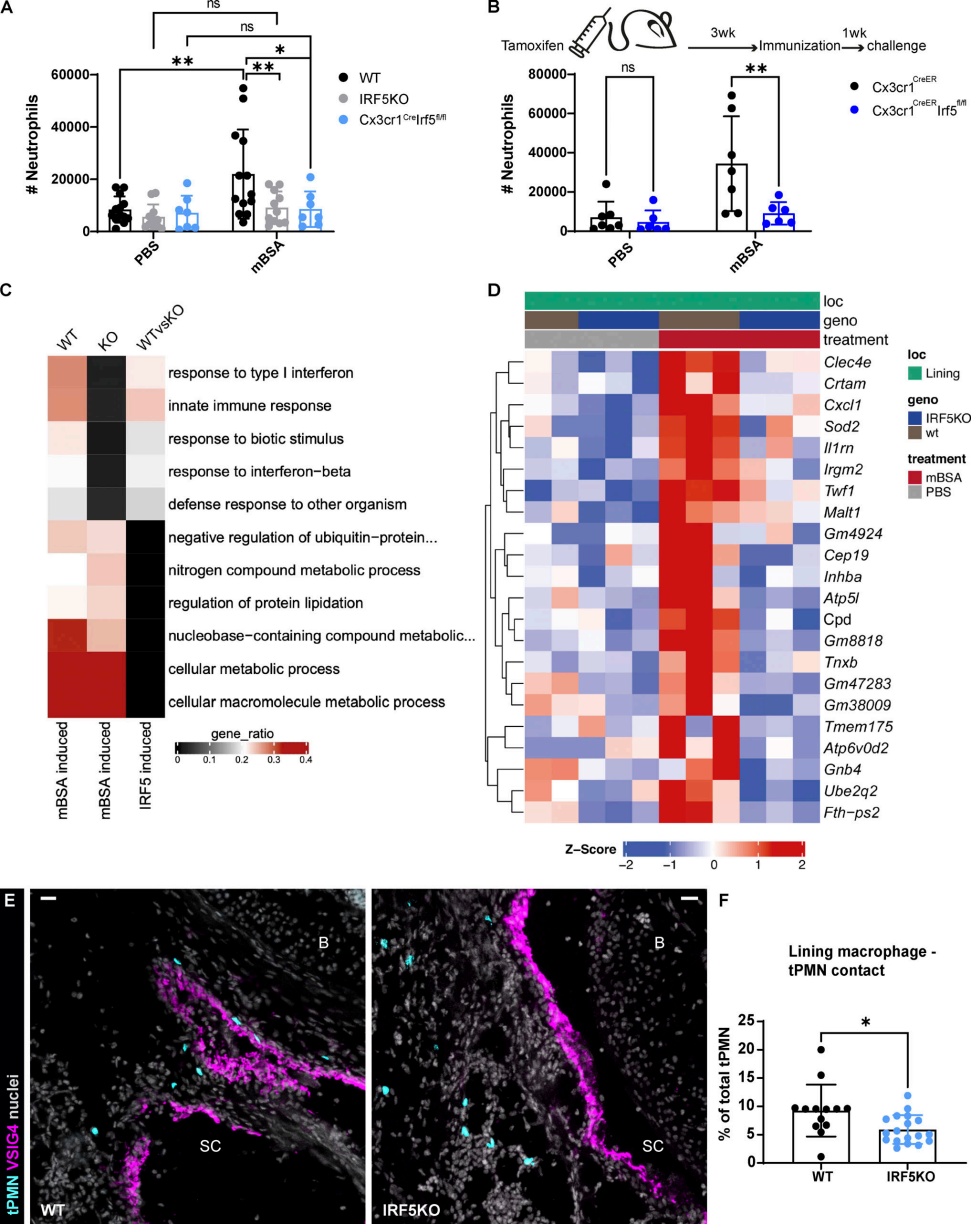
**IRF5 deficiency impairs the activation of lining macrophages and neutrophil recruitment at the onset of AIA. (A)** FACS assessment of synovial neutrophil numbers at the onset of AIA (6 h) demonstrates diminished recruitment in both global and CX3CR1 driven IRF5KO mice. Data were pooled from three independent experiments with four to six mice per group. P values by two-way ANOVA with Šídák's multiple comparisons post hoc: *, P < 0.05; **, P < 0.01. **(B)** Reduced synovial neutrophil counts at 6 h p.c. by FACS in lining macrophage–specific IRF5KO (CX3CR1^CreER^IRF5^fl/fl^) achieved by tamoxifen administration 4 wk prior to induction of AIA. P values from a single experiment by two-way ANOVA with Šídák's multiple comparisons post hoc: **, P < 0.01. **(C)** GO comparison of WT and IRF5KO lining macrophages shows impaired activation of IRF5KO cells in response to mBSA 4 h p.c. **(D)** Differentially expressed genes between WT (wt) and IRF5KO lining macrophages at 4 h after mBSA challenge, with CXCL1 expression markedly reduced in IRF5KO cells. **(E)** Representative confocal images of the synovial lining in WT and IRF5KO mice receiving adoptive transfer of WT neutrophils reveals that transferred neutrophils interact more with lining macrophages in WT mice compared to IRF5-deficient mice at 4 h p.c. (transferred neutrophils [tPMN], cyan; VSIG4, magenta; nuclei, gray; scale bar = 30 µm). **(F)** Quantification of the contact between lining macrophages and transferred neutrophils from a single experiment (*N* = 2–3; *n* = 13–18). *N* indicates the number of mice per group, *n* the number of total sections quantified. P value by Wilcoxon rank–sum test: *, P < 0.05. B, bone; SC, synovial cavity.

Mice systemically lacking IRF5 accumulate far fewer neutrophils in the joint due to the reduced levels of CXCL1 (Weiss et al., 2015). Lining macrophages express IRF5 to a higher level than both Ly6C^+^ monocytes and neutrophils at the onset of AIA (Fig. S3 B). As CX3CR1 expression specifically marks lining macrophages in naive animals (Fig. S1, A and B), we crossed the CX3CR1-Cre and CX3CR1-CreER reporter mice with IRF5^fl/fl^ mice to examine the hypothesis that IRF5 ablation in lining macrophages would phenocopy the global IRF5 deficiency at the onset of inflammation. The CX3CR1^cre^IRF5^fl/fl^ mice lacked IRF5 preferentially in lining macrophages, but its potential role in recruited monocytes expressing CX3CR1 could not be excluded. The CX3CR1^CreER^IRF5^fl/fl^ mice were challenged with mBSA 4 wk after tamoxifen administration, ensuring that IRF5 was removed only in lining macrophages without affecting its levels in recruited monocytes. We observed lower synovial neutrophil counts compared with the control mice with either global or lining macrophage/monocyte–, or lining macrophage–specific IRF5 deletion at the onset of AIA measured by flow cytometry (Fig. 5, A and B) or confocal microscopy (Fig. S3 C). Thus, our data demonstrate that lining macrophage–specific IRF5 activity controls initial inflammatory response in the synovium. Moreover, lining macrophage–specific deletion of IRF5 leads to improved histopathology scores at the resolution of inflammation, assessed by Fast Green and Safranin O staining on knee sections on day 7 p.c. for the synovial hyperplasia, bone erosion, and BM proliferation (Fig. S3, D and E), indicating that IRF5-meditated activation of these cells has lasting consequences in this model of articular inflammation.

Next, we compared transcriptomes of WT and IRF5-deficient lining macrophages at 4 h p.c. by small bulk RNA sequencing. IRF5-deficient lining macrophages displayed less inflammatory molecular signature in response to mBSA compared with the WT lining macrophages (Fig. 5 C). Furthermore, *CXCL1* gene expression upon mBSA challenge was significantly decreased in IRF5-deficient lining macrophages compared with WT lining macrophages (Fig. 5 D). When we adoptively transferred fluorescently labeled WT neutrophils i.v. into WT mice 3 h after mBSA challenge and examined their position within the knee at 4 h p.c., we found that transferred neutrophils localized near VSIG4^+^ areas in the synovial lining (Fig. 5 E). In contrast, neutrophils transferred into challenged IRF5KO hosts failed to associate with these structures and were instead located in sublining regions (Fig. 5 E). To quantify these positional patterns, we determined the direct contact between the neutrophils and lining macrophages by measuring the distance between these two cell types, contact being defined as zero distance. Our analysis revealed that IRF5-deficient lining macrophages formed less contact with the transferred neutrophils compared with WT lining macrophages (Fig. 5, E and F), confirming that IRF5-controlled mediators (i.e., CXCL1) produced by lining macrophages are important for neutrophil extravasation and clustering in the lining.

### Concluding remarks

Our study highlights lining macrophages as critical upstream instigators of joint inflammation. This is seemingly in contrast with published observations which describe lining macrophages in the context of their immunosuppressive functions; however, most of these studies, especially in humans, focused on isolated cells and/or relatively late time points of the disease, thereby overlooking the initial entry site of neutrophils into the synovial tissue and the role of lining macrophages in mediating this process. Focusing on the early hours post mBSA challenge, we demonstrate for the first time that lining macrophage activation leads to the inflammatory response dominated by neutrophil recruitment into the lining. Investigating early activation of immune response in the human synovium is challenging as clinical disease manifestations of RA occur years after its pathogenesis has long begun. However, understanding the initiation phase of RA can inform early therapy approaches, improving long-term outcome of this disease.

In this study, we demonstrate that lining macrophages produce neutrophil-recruiting chemokines as a result of phagocytosis of antigen–antibody complexes. By challenging mice with an antigen (PE) or PE-specific antibodies, we show that lining but not sublining macrophages preferentially uptake ICs and that the ingested amount directly correlated with synovial neutrophil counts. Interestingly, in the lymph nodes and the spleen, subcapsular and marginal zone lining macrophages, respectively, are the main cell types that capture and initiate immune responses to ICs that are drained into the tissue from the lymph and the blood (Batista and Harwood, 2009; Cyster, 2010). Noteworthy, formation of ICs also occurs in AIA (Cooke et al., 1983), in which we observe phagocytosis of labeled mBSA, which leads to macrophage activation and neutrophil recruitment to the lining. In another mouse arthritis model, serum transfer–induced arthritis, a lipid–cytokine–chemokine cascade controlled in a spatial and temporal manner underlies neutrophil recruitment into the synovium (Chou et al., 2010), and the initial immune activation involves the deposition of ICs onto the cartilage and complement activation (Miyabe et al., 2017). The recognition of ICs may be an early immune-activating event in humans as well, as ICs can be covalently coupled to hyaluronate and retained within the synovial cavity (Prehm, 1995), potentially activating lining macrophages. In human arthritis, where neutrophils are abundant in the synovial fluid (Asif Amin et al., 2017), IC-activated lining macrophages may control neutrophil entry into the synovial cavity from the lining area.

The activation of lining macrophages does not happen in isolation. On the contrary, we observed induction of CXCL1 in other cells in the lining vicinity, including the newly recruited neutrophils. Thus, we hypothesize that lining macrophages induce expression of CXCL1 in what we term “synovial lining niche.” Alternatively, other mechanisms independent of lining macrophages may account for CXCL1 topography, including direct recognition of mBSA complexes by the lining fibroblasts and newly recruited neutrophils. Methylated BSA can be recognized by NLRP3 inflammasome and activate the secretion of IL-1β (Di Domizio et al., 2013), which may in turn stimulate the production of CXCL1 (Wen et al., 1989; Anisowicz et al., 1991). Nevertheless, given that specific inactivation of IRF5 in lining macrophages leads to significantly reduced neutrophil recruitment, they seem to be important regulators of inflammation at the onset of AIA. Our observations, together with other RA studies focusing on different cells, such as monocytes (Misharin et al., 2014) or fibroblasts (Croft et al., 2019), indicate that cell cooperation may be necessary for disease development. Our approach to studying joint inflammation in situ presents an important avenue for gaining better understanding of cell communication and function tied to anatomical position in the context of pathophysiology.

## Materials and methods

### Mice

C57BL/6J and IRF5KO (*Irf5*^*tm1Ttg*^) mice were used for small bulk RNA sequencing, FACS, and imaging experiments. Conditional macrophage–specific IRF5KO (cIRF5KO, IRF5-LoxP/LoxP, CX3CR1-Cre/Cre: *Irf5*^*tm1Ppr*^ hom, *Cx3cr1*^*tm1.1(cre)Jung*^ hom) and control CX3CR1-cre (*Cx3cr1*^*tm1.1(cre)Jung*^ hom), as well as tamoxifen-inducible CX3CR1^CreER^IRF5^fl^ (*Cx3cr1*^*tm2.1(cre/ERT2)Jung/J*^ hom, *Irf5*^*tm1Ppr*^ hom) mice were used in FACS experiments. CX3CR1-eGFP Ly6G-tdTomato double reporter mice (*Cx3cr1*^*tm1Litt*^/+, *Ly6g*^*tm2621(Cre-tdTomato)Arte*^/+, *Gt(ROSA)26Sor*^*tm14(CAG-tdTomato)Hze*^/+) were used in imaging AIA time course. In some imaging experiments, CX3CR1-eGFP IRF5KO (CX3CR1-gfp/+, IRF5^−/−^) and CX3CR1-eGFP (CX3CR1-gfp/+, IRF5^+/+^) were used. Genetically modified mice were on C57BL/6J background. All experimental mice were between 8 and 12 wk old. The animals were bred and maintained in specific pathogen–free conditions in the Kennedy Institute of Rheumatology animal facility. All experiments were approved by the Clinical Medicine Animal Welfare and Ethical Review Board and the UK Home Office, in accordance with the 1986 Animals (Scientific Procedures) Act.

### AIA

Induction of joint inflammation was performed by a modified version of the previously described method (Brackertz et al., 1977). Briefly, mice were immunized by two subcutaneous injections of 20 µg mBSA (40 mg/ml) emulsified with CFA (3.3 mg/ml) and PBS to a final 100 μl volume per mouse. The emulsion was prepared using BTB immunization kit (BTB Emulsions AB). The injections were performed under isoflurane anesthesia, one at the base of the tail and the other at the right flank, 50 μl/each. 7 d after, knee inflammation was induced by IA injection of 200 µg mBSA (10 μl of 20 mg/ml stock) using Hamilton 50 μl syringe with Luer tip (Scientific Laboratory Supplies Limited), whereas contralateral knee received 10 μl PBS as a control. IA injections were performed under isoflurane anesthesia.

### Labeling of mBSA

mBSA was labeled with NHS-Alexa 555 kindly provided by a collaborator from the Nuffield Department of Medicine in Oxford. Briefly, 3 μl of dye ester at 1 mg/ml was used per 10 μl (20 mg/ml) of mBSA to reach ∼1% degree of labeling. The reaction was performed in equal volume 25 mM Tris buffer. The protein was first incubated for 10 min in Tris buffer and the dye was subsequently added. The reaction was allowed to occur for 45 min at room temperature. Subsequently, sample concentration and the removal of unbound dye were done by ultrafiltration using VivaSpin 500 column, 10,000 molecular weight cut-off (Sartorius), observing the manufacturer’s recommendations.

### Adoptive transfer of neutrophils

Five WT male mice were used as donors for neutrophil isolation and transfer into two syngeneic IRF5 sufficient recipients (CX3CR1-gfp/+, IRF5^+/+^) and three syngeneic IRF5 deficient recipients (CX3CR1-gfp/+, IRF5^−/−^). Bone marrow of donor mice was isolated by centrifugation, according to a published protocol (Amend et al., 2016). The resulting bone single-cell suspension was labeled with 10 µM CellTrace Far Red (Life Technologies) for 20 min at room temperature, protected from light. Any free dye was removed by a 5-min incubation with complete RPMI 1640, and the suspension was spun down to pellet the cells. The cells were then subjected to negative neutrophil isolation using Neutrophil Isolation kit (Milteny), following the manufacturer’s protocol. Magnetic separation was carried out using LS columns and QuadroMACS manual separator (Milteny). Purified neutrophils were resuspended in sterile PBS, and roughly 2 million neutrophils were injected i.v. into each recipient mouse 1 h following challenge with mBSA. Recipient mice were sacrificed at 4 h after challenge (3 h after neutrophil transfer) and the knees were isolated and processed for confocal microscopy as described below.

### Generation of transgenic T cells

For CXCR2 overexpression, CXCR2 sequence was cloned into the murine stem cell virus (MSCV2.2) retroviral vector followed by an internal ribosomal entry site and a Thy1.1 reporter sequence to generate MSCV-CXCR2-IRES-Thy1.1 vector. As a control, empty MSCV-IRES-GFP vector was used.

To generate viral vector particles, PLAT-E packaging cell line was grown in DMEM supplemented with 10% FBS, Hepes, and penicillin–streptomycin. At 60% confluency, a 10-cm dish of PLAT-E cells was washed with PBS and antibiotic-free medium was added before transfection solution of 1400 μl Opti-MEM containing 10 μg MSCV plasmid vector and 20 μl Lipofectamine 2,000 (11668019; Invitrogen) was added dropwise to the plate. 24 h after transfection, the medium was exchanged with 7 ml fresh PLAT-E medium, which was collected 24 h later, 0.45 μm filtered, supplemented with 4 μg/ml polybrene, Hepes, and IL-2 AT 5 ng/ml (200-02-100uG; Peprotech), and used for transduction of activated T cells.

To activate T cells, 2 d prior to transduction, splenocytes were plated at 5 × 10^6^ cells/well in T cell medium (RPM1640 supplemented with 10% FBS, Hepes, penicillin–streptomycin, 2-mercaptoethanol) supplemented with a-CD28 at 2 μg/ml (102116, clone 37.51; BioLegend) in 24-well plates precoated with a-CD3 (100340, clone 145-2C11; BioLegend). On day 2 after activation, cells were split at 1:3 to 1:5 in a fresh 24-well plate and the T cell medium was exchanged with 1 ml/well vector-containing PLAT-E medium. Cells were spun at 1,400 *g* with no break for 2 h at 35°C. T cells were split every 2 d in IL-2 supplemented T cell medium and kept for up to 2 wk before injection.

### Adoptive transfer of transgenic T cells

Suspension containing several million CXCR2 OE T cells was labeled with 3 µM CellTrace Yellow (Life Technologies) for 20 min at room temperature, protected from light, whereas equal number of control T cells were labeled with CellTrace Far Red. Any free dye was removed by a 5-min incubation with complete RPMI 1640, and the suspension was spun down to pellet the cells. Control and CXCR2 OE cells were resuspended in sterile PBS, counted, and combined in 1:1 ratio. 10 million of each control and CXCR2 OE cells (20 million in total in 100 μl) were i.v. injected into recipient mice 1 h after AIA induction. Mice were sacrificed 6 h p.c. (5 h after T cell transfer) to collect blood and knee joints for FACS and confocal microscopy.

### Formation and uptake of ICs

To assess the uptake of ICs by synovial resident macrophages, we have (1) passively immunized mice against PE by injecting 300 µg of rabbit-derived anti-PE immunoglobulins i.v. (Invitrogen) in 100 μl volume, 3 h prior to IA challenge, followed by IA PE administration of 12 µg of PE fluorochrome (Invitrogen) or PBS control (10 μl). A separate cohort of mice was (2) injected with PE alone into the knee, without prior passive immunization, whereas contralateral knee was (3) challenged with the preformed PE ICs (1:10 stoichiometry: 12 µg PE + 120 µg anti-PE Ab in 10 μl, incubated for 15 min prior to injections). PE fluorescence in macrophages and resulting neutrophil recruitment were analyzed 3 h after IA injections by FACS of the cells of disaggregated synovia. Further, knees from two mice of each cohort were prepared for confocal microscopy as described below.

### Tamoxifen administration

We administered 4 mg of tamoxifen by intraperitoneal injection into the CX3CR1^CreER^IRF5^fl/fl^ mice and CX3CR1^CreER^ controls 4 wk prior to the induction of AIA. The injections were performed on three consecutive days with 1 mg of tamoxifen administered on the first day and 1.5 mg on following 2 d. Mice were immunized 3 wk after the last tamoxifen dose and challenged with mBSA a week later to induce synovial inflammation. This regimen allowed us to bypass the effect of tamoxifen on CX3CR1 expressing monocytes due to their rapid turnover and to efficiently target longer-living lining macrophages. Tamoxifen was prepared by dissolving in corn oil at 10 mg/ml using an ultrasonic bath.

### Synovial isolation and single-cell suspension

Isolation of the synovium was carried out by the adaptation of the previously published protocol (Van Meurs et al., 1997). Mice were euthanized with CO_2_ and the skin of the lower limbs was dissected to expose the muscles. Horizontal incision traversing biceps femoris anterior and posterior, severing patellar ligament, as well as adductor longus and vastus medialis muscles, was made using small surgical scissors. Excess muscle covering femur (rectus femoris, vastus lateralis) was dissected and a circular cut was made connecting to the horizontal incision (an interactive view on the anatomy of mouse hind limbs; Charles et al., 2016). This allowed the removal of patella and exposure of the fat pad for further dissection, which was carried out using stereo microscope. Fat pad was isolated by careful dissociation from the lateral and cruciate ligaments, using fine-toothed forceps. Isolated tissue (patella together with surrounding muscle and periosteum, as well as the fat pad) was kept on ice in 1 ml of RPMI 1640 without FCS and supplemented with 1% penicillin/streptomycin until further processing. Single cells were obtained by incubating the tissue at 37°C for 1 h in 2 ml DNAse I (1 mg/ml, Roche) and Liberase TL (0.2 mg/ml, Roche) in FCS-free RPMI 1640, lightly shaking. The suspension was passed through a 70-µm strainer, and the leftover tissue was mechanically disrupted using the plunger of a 5 ml syringe, followed by a rinse with 5 ml of FCS-free RPMI.

### Flow cytometry and cell sorting

For the analysis of synovial cell composition using extracellular markers, single cells were transferred into 96-well polypropylene U bottom plates and stained with 1 µM Live/Dead Fixable Near-IR dye (Thermo Fisher Scientific) for 20 min in PBS at 4°C. Unbound dye was removed by washing with PBS and cells were incubated with 10 μl of purified rat anti-mouse CD16/CD32 Fc block (BD Biosciences) diluted 100× in FACS buffer containing PBS, 0.1% BSA, and 2 mM EDTA to block unspecific staining. Cells were labeled with fluorochrome-conjugated antibodies diluted 1/100 in FACS buffer (20 μl/sample) for 30 min at 4°C, protected from the light. Cells were washed, fixed with BD Cytofix (BD Biosciences) according to the manufacturer’s protocol, and resuspended in FACS buffer prior to acquisition. BD CountBrite beads (10 μl, BD Biosciences) were added to each sample to estimate total cell numbers. Samples were acquired on BD LSR Fortessa and analyzed using FlowJo v10 Software.

Sorting of synovial macrophages was carried out from the knee cell suspensions stained as described above. Lining macrophages were defined as CD45^+^, Linage^−^ (NK1.1, CD3, CD19), CD11b^+^, F4/80^+^, and VSIG4^+^ cells, whereas sublining macrophages were CD45^+^, Lin^−^, CD11b^+^, F4/80^+^, and VSIG4^−^ (Fig. S1 B). 200 macrophages were sorted through a 70-µm nozzle directly into 2.2 μl of lysis buffer (composition detailed below) using BD FACS Aria III.

Intracellular staining of IRF5 was performed using FoxP3/Transcription Factor Staining Buffer Set (eBiosciences) according to the manufacturer’s protocol. Cells were incubated with anti-IRF5 antibody (Abcam) diluted 1/200 in Perm/Wash buffer (20 μl/sample) for 30 min at 37°C, protected from the light, followed by the goat anti-rabbit secondary antibody conjugated to Alexa Fluor 555 (1/500; Life Technologies). Table S1 lists all antibodies used in FACS and sorting experiments.

### Small bulk RNA sequencing and data analysis

200 lining and sublining macrophages from three WT and three IRF5KO mice, isolated from PBS and mBSA injected knees, were sorted into PCR tubes containing 2.2 μl lysis buffer (0.8% [vol/vol] Triton X-100 and 2 U/μl RNase inhibitor [Clontech]), according to the above-described gating, shown in Fig. S1 B. Samples were subsequently transferred into 96-well skirted PCR plate, sealed with an adhesive PCR plate seal (Thermo Fisher Scientific), and transferred to Oxford Genomics Centre on dry ice to be sequenced. Briefly, the preparation of libraries was done using NEBNext Ultra-low/single cell (Smarter-like) protocol (New England BioLabs). The libraries were size-selected, multiplexed, and quality-checked, followed by paired-end sequencing over two units of a flow cell using Illumina NovaSeq 6000 system. Data were aligned to the reference and quality checked. Resulting data were mapped to the mm10 genome using STAR v2.7.5 b with the options: --runMode alignReads--outFilterMismatchNmax 2. Uniquely mapped read pairs were counted over annotated genes using featureCounts v.2.0.0 with the options: -T 18 -s 2 -Q 255. Differential expression was then analyzed with DESeq2 v1.24.0 (Love et al., 2014). Variance-stabilized counts for all DESeq2 differentially expressed genes, likelihood ratio test, and false discovery rate < 0.01 were used for dimensionality reduction and the heatmaps. GO analysis was performed using one-sided Fisher’s exact tests, as implemented in the gsfisher v0.2 R package (https://github.com/sansomlab/gsfisher/).

### Preparation of mouse knees for confocal microscopy

Mouse knees were excised by severing femur and tibia ∼3 mm from the joint and excess muscle was removed. The knees were fixed in 5 ml PLP buffer (McLean and Nakane, 1974) containing PBS, 1% methanol-free PFA, 1.3% wt/vol L-lysine, and 0.2% wt/vol sodium metaperiodate at 4°C overnight under light shaking conditions. The samples were then briefly washed with PBS and decalcified at 4°C, shaking in 10 ml of 0.5 M EDTA, pH adjusted to below 8 for 5–6 d. The solution was exchanged twice in the process. Following decalcification, the samples were incubated in 10 ml 30% sucrose for 24 h at 4°C. The embedding of the knees was carried out according to the published protocol (Kusumbe et al., 2015). Briefly, the samples were incubated in embedding solution of 8% gelatin (from bovine skin, type B, Sigma-Aldrich), 20% sucrose, and 2% polyvinylpyrrolidone (5 ml) in a 60°C water bath for 1 h and transferred to 15 × 15 × 5 mm Tissue-Tek Cryomold molds (Agar Scientific). Gelatin was allowed to polymerize (30 min) and molds were then frozen in a dry ice–methanol slurry and transferred at −80°C.

### Immunofluorescence staining of frozen knee sections

Frozen knee blocks were transferred to Leica CM3050 cryostat set to −28°C to equilibrate for 30 min–1 h before sectioning. Subsequently, 20-µm-thick sections were cut and three consecutive sections were placed on each gelatin-coated slide. Sections were allowed to air-dry for 3 h before being stored away at −20°C.

For immunostaining, sections were allowed to air-dry for 40 min–1 h followed by a 20-min incubation in a 65°C oven, vertically placed in a staining tub. ImmEdge Hydrophobic Barrier PAP pen (Vector Laboratories) was applied to the slides to keep reagents from running and sections were rehydrated with 500 μl PBS per slide. Slides were then incubated with Carbo-free blocking solution (Vector Laboratories) supplemented with 0.05% Tx-100, 0.08% sodium azide, and 0.3 M glycine for 1 h at room temperature to block unspecific antibody binding. In some instances, Image-iT FX Signal Enhancer (Life Technologies) was applied to the sections following incubation with a blocking buffer, observing the manufacturer’s recommendations. Sections were stained with primary antibodies either overnight at 4°C or for 1 h and 30 min at room temperature, whereas secondary antibodies were always applied at room temperature for 1 h and 30 min. The antibodies were diluted in Carbo-free blocking solution containing only 0.08% sodium azide, and 400 μl staining solution was applied per slide. Primary antibodies were always diluted to apply a total of 1 µg per slide, whereas 2 µg of secondary antibodies was used. The antibodies used for immunostaining are listed in Table S1. Washing steps were performed in PBS (three times for 5 min) and nuclei were counterstained with Sytox Blue (Life Technologies), 0.2 µM in 400 μl PBS with 0.05% Tx-100 for 20 min at room temperature. Slides were briefly rinsed by immersion in deionized water and excess liquid was removed using lint-free tissue. FluorSave (Life Technologies) mounting medium was used to place 22 × 50 mm coverslips while avoiding bubble formation. Slides were cured for 1 h or overnight before imaging.

### Coating of slides with gelatin

SuperFrost Plus glass slides (VWR International Ltd.) were coated with a gelatin-coating solution consisting of 5 g gelatin and 0.5 g chromium potassium sulfate dodecahydrate CrK(SO_4_)_2_ × 12H_2_O (Alfa Aesar) per liter of deionized water. Briefly, gelatin was dissolved in deionized water in a 60°C water bath followed by the addition of CrK(SO_4_)_2_. The slides were placed into metal racks and dipped into the coating solution three to five times (5 s each). The racks were drained and blotted using a paper roll and left to dry for 48 h at room temperature, protected from dust, or overnight in a 37°C incubator. Slides prepared in such a way were kept at room temperature until sectioning. Leftover gelatin can be stored at 4°C for a few weeks.

### Microscopy

#### Fluorescent markers

For AIA time-course imaging, CX3CR1-eGFP Ly6G-tdTomato mice were used. Briefly, rabbit-derived anti-GFP primary antibody (Life Technologies) followed by the goat anti-rabbit Alexa Fluor 488 (Life Technologies) was used to enhance CX3CR1 detection. Total macrophages were labeled with either unconjugated rat anti-CD68 (Bio-Rad) and goat anti-rat Brilliant Violet 421 secondary antibody (BD Biosciences), or anti-CD68 directly conjugated to Brilliant Violet 421 (BD Biosciences), whereas goat-derived anti-VSIG4 (Bio-Techne) followed by donkey anti-goat Alexa Fluor 647 (Life Technologies) was used to delineate lining macrophages. Neutrophils were detected based on the expression of tdTomato. In part of the mBSA uptake experiments, in addition to the aforementioned macrophage markers, rabbit CD177 (Bio-Techne) was used with goat anti-rabbit in Alexa Fluor 488 (Life Technologies) to mark neutrophils. Examination of CXCL1 in situ was accomplished using rabbit anti-CXCL1 Alexa Fluor 647 (Bio-Techne) boosted by donkey anti-rabbit in Alexa Fluor 647 Plus (Life Technologies). In this case, secondary antibody for VSIG4 was donkey anti-goat in Alexa Fluor 594 (Abcam), whereas endothelium was marked with primary rat anti-CD31 (BD Biosciences) and the secondary goat anti-rat in Alexa Fluor 488 (Life Technologies); anti-CD68 Brilliant Violet 421 (BD Biosciences) was used for macrophages. To examine endothelial activation, slides were stained with rat CD62E (BD Biosciences) followed by goat anti-rat Alexa Fluor 488, rat CD31 directly conjugated to Alexa Fluor 647 (BioLegend), rabbit Prg4 (Abcam) with goat anti-rabbit Alexa Fluor 594 (Abcam), and finally anti-CD68 Brilliant Violet 421. In the neutrophil transfer experiment, slides were stained with VSIG4 and donkey anti-goat Brilliant Violet 421 (Stratech Scientific Ltd.), as well as CD177 followed by the goat anti-rabbit Alexa Fluor 555 (Life Technologies). Transferred neutrophils labeled with CellTrace Far Red were detected in red emission region (636–758 nm). In T cell transfer experiment, sections were stained with VSIG4 and donkey anti-goat Brilliant Violet 421 (Stratech Scientific Ltd.), as well as CD177 followed by the donkey anti-rabbit Alexa Fluor 488 (Life Technologies) and CD31 directly conjugated to Alexa Fluor 594 (BioLegend). Transferred T cells labeled with either CellTrace Far Red (control) or CellTrace Yellow (CXCR2 OE) were detected in red (636–758 nm) and yellow (550–615 nm) emission regions, respectively. Nuclei were always counterstained with Sytox Blue.

#### Image acquisition and processing

Images were acquired on Zeiss 880 laser scanning confocal system on an Axio Examiner upright fixed stage microscope or Zeiss 980 laser scanning confocal inverted microscope equipped with an Airy Scan detector. For imaging AIA time course as well as some endothelial activation and mBSA uptake experiments, Plan-APO 10×/0.45 objective was used, whereas examination of CXCL1 in situ distribution, part of E-selectin staining experiments, as well as other mBSA uptake experiments, were carried out with Plan-APO 20×/0.8 objective. Precise cellular localization of CXCL1 was inspected on Zeiss 980 confocal microscope using Plan0-APO 63×/1.4 objective. Entire knee cross-sections were acquired using a tile scan function with 10% overlap, sampling at half Nyquist criterion, frame-wise, with pinhole size ranging from 1 to 1.45 airy units. In some cases, frames were acquired twice and the resulting signal was averaged. Z-stacks were acquired with optical section thickness overlapping by ∼1%. Excitation of Brilliant Violet 421 was done with 405 laser line, Sytox Blue was excited with 458 laser, Alexa Fluor 488 with 488 laser, labeled mBSA and Alexa Fluor 555 with 561 laser, Alexa Fluor 594 with 594 laser, whereas Alexa Fluor 647 was excited using 633 laser. Fluorescence signals were acquired with sequential scans using point detection, set by observing maximum emission and minimizing fluorescence crossover between individual fluorochromes. Tiled images were stitched and orthogonal maximum intensity projections were generated from z-stacks after acquisition in Zen Blue v2.3 software. All images used in figures were generated using Imaris v9.8.0 software (Oxford Instruments) with brightness adjusted to allow for optimal visualization of distinct image features.

### Image analysis

#### mBSA uptake

Region of interest (ROI) representing the whole anterior part of the knee cross-section was manually drawn in Imaris software using “Surfaces” function. Fluorescent signals representing lining and sublining macrophages within that ROI were then duplicated for further segmentation of the image. Lining macrophages were segmented as surfaces based on the fluorescent intensity of the VSIG4 signal. Briefly, signal threshold was determined manually, and touching surfaces weren’t separated. Sublining macrophages were similarly segmented based on the intensity of CD68 staining with an additional filter in VSIG4 channel applied to separate them from the lining macrophages. Namely, only macrophages that had a pixel value below a certain manually set threshold in VSIG4 channel were counted as sublining macrophages. mBSA signal from within the resulting lining and sublining macrophage surfaces was extracted (“Mask channel” tool within “Surfaces” function) and segmented again using “Surfaces” function, according to mBSA fluorescent intensity, setting the threshold for “positivity” manually and taking care to use the same threshold within both cell types. A ratio of intramacrophage mBSA surface area and total macrophage surface area, expressed as a percentage, was compared for the lining and sublining macrophages.

#### Neutrophil distribution

As with the quantification of mBSA uptake, ROI across the whole anterior part of the knee cross-section was manually drawn in Imaris with the “Surfaces” function, and fluorescent signal representing neutrophils within that region was extracted using “Mask channel” tool. Neutrophils were segmented as surfaces based on a manually set threshold for fluorescence intensity of tdTomato, and separation of touching surfaces was enabled, as it was important to segment each individual neutrophil. The lining was manually drawn at the border of synovial tissue and joint cavity using “Filament” function and adjusted based on VSIG4 or Prg4 staining where possible. The lining filament was converted into a separate channel and lining surface was created out of it. “Distance transformation” tool in the lining surface was used to measure the distance of neutrophil surfaces to the lining surface. Neutrophil distances on every section were normalized by the distance value of the furthest cell on that section, which allowed combining several sections of the same knee/mouse, as well as comparison of different mice and time points.

#### CXCL1 distribution

Similarly to the above, CXCL1 signal was extracted from the manually drawn synovium ROI with the “Mask channel” tool and segmented as a surface based on a manually set threshold for fluorescence intensity in its respective channel, with separation of touching surfaces function enabled. Synovial lining was segmented as a surface as described above. “Distance transformation” tool in the lining surface was used to measure the distance of CXCL1 surfaces to the lining surface. The distance relationship between the lining and CXCL1 surfaces was plotted using the Vantage function in Imaris.

#### Endothelial activation

As described above, manually drawn synovium ROI was generated from the anterior part of the knee cross-section. Using the “Mask channel” tool, CD31 signal was extracted and segmented as a surface based on a manually set threshold for fluorescence intensity in its respective channel, with splitting of touching surfaces enabled. Further, in cases where there was extensive autofluorescence in CD31 channel coming from the fat present in the tissue, the surfaces were manually edited to remove non-signal, i.e., autofluorescent parts. Thus, segmented vasculature surfaces were filtered based on the intensity of E-selectin (CD62E) staining to identify signal threshold above which there are between 5 and 15% of the “brightest” vessels. Whether top 5% or 15% CD62E expressing blood vessels were observed depended on the amount of CD62E^+^ staining on a particular section. This signal threshold/fluorescence intensity value was used to classify the surfaces in CD62E^−/low^ and CD62E^+/high^. Synovial lining was segmented as in previous examples and the “Distance transformation” tool in the lining surface was used to measure the distance of each blood vessel surface to the lining surface. The distance between the lining and CD62E^−/low^, as well as CD62E^+/high^ surfaces was plotted using Vantage function in Imaris.

#### Lining macrophage–neutrophil contact

ROI consisting of both anterior and posterior synovium was drawn, and VSIG4 channel as well as transferred neutrophil channel were duplicated within it for further segmentation. Lining macrophages were masked as surface based on the VSIG4 channel, as described above, with “Object to object statistic” functionality included in the masking algorithm. Transferred neutrophils were similarly segmented based on their respective channel of fluorescence using “Surfaces” with “Object to object statistic” enabled. Neutrophil surfaces that had 0 µm distance from the lining macrophage surface were counted as being in direct contact with each other.

#### mBSA-laden lining macrophage interaction with neutrophils

ROI consisting of anterior synovium was drawn and VSIG4 and neutrophil channel were duplicated within the ROI for further segmentation. Lining macrophages were masked as surfaces based on the VSIG4 channel, as described above, with “Object to object statistic” functionality included in the masking algorithm. mBSA signal within lining macrophage surfaces was extracted and segmented into a separate surface. Lining macrophage surfaces were classified into mBSA^low/int^ and mBSA^high^ based on the amount of overlap with mBSA surface (40% overlap and above was labeled as mBSA^high^ lining macrophage). Neutrophils were similarly segmented based on their respective channel of fluorescence using “Surfaces” with “Object to object statistic” enabled. Degree of interaction between classified lining macrophages and neutrophils was measured as an overlap of their respective surfaces, normalized by the total surface of a lining macrophage.

### Data visualization and statistics

Synovial neutrophil distribution is presented in the form of violin plots, indicating population medians and interquartile range. Kolmogorov–Smirnov test was used to calculate statistical significance. FACS assessment of synovial neutrophil counts is presented using a scatter plot, with means and SD indicated. Data distribution in the FACS neutrophil assays was examined using two-way ANOVA with Šídák's multiple comparisons post hoc test and by one-way ANOVA and Šídák multiple comparisons in IC experiment. IC uptake was represented as geometric mean of PE fluorescence in VSIG4^+^ lining and VSIG4^−^ sublining macrophages using a scatter plot, with means and SD indicated and groups compared using mixed-effects analysis and Šídák multiple comparisons as post hoc. The number ratio of T cells in adoptive T cell transfer experiment is presented by a scatter plot, lines connecting tissue compartments of the same mouse which were compared using paired *t* test. The contact of transferred neutrophils to lining macrophages was visualized with a scatter plot, mean ± SD, and analyzed with Mann–Whitney test. Individual values are also presented in mBSA uptake quantification, lines connecting lining and sublining macrophages of the same sample, and Wilcoxon-matched-pairs signed rank test was used to calculate significance. These data were plotted and analyzed using GraphPad Prism v9.2.0 (GraphPad Software, Inc). The pattern of CXCL1 and CD62E expression was visualized using Vantage plots in Imaris v9.8.0, demonstrating individual values together with medians and interquartile ranges. The distribution of activated (CD62E^+/high^) blood vessels to non-activated vessels was compared with the Wilcoxon rank-sum test. Probability values (P) <0.05 were considered significant. *, P < 0.05; **, P < 0.01; ***, P < 0.001; and ****, P < 0.0001.

Group size for synovial neutrophil distribution was three mice for the onset of inflammation (6 h), three mice for day 2 and day 7 after challenge, with two to three sections at different knee z positions (sectioned in sagittal) per mouse. The uptake of mBSA study was performed twice independently and images from a single representative study were quantified (two mice, 4 and 6 h p.c., six different sections each). CXCL1 study was done on five mice, examining two sections per mouse. CD62E study was performed on four mice, quantifying two to four knee sections per mouse. FACS of the synovium from WT, IRF5KO CX3CR1^Cre^IRF5^fl/fl^ mice was performed three times, with four to six mice per group, and resulting data were pooled. Synovial inflammation assessment by FACS on CX3CR1^CreER^IRF5^fl/fl^ and CX3CR1^CreER^ control mice treated with tamoxifen was performed on six vs. seven mice, respectively, in a single experiment. Small bulk RNA sequencing was performed on lining and sublining macrophages from both PBS and mBSA injected knee from three WT and three IRF5KO mice. Two WT and three IRF5KO recipients were used in the neutrophil transfer study, and the examination of cell contact with lining macrophages was undertaken on 13 vs. 18 different knee cross-sections, respectively. Adoptive T cell transfer was undertaken on three recipient mice for FACS assessment and two mice for confocal microscopy. Images of the adoptive T cell transfer shown in Fig. 4 are representative of four different sagittal knee cross-sections acquired from each mouse.

### Online supplemental material

Fig. S1 shows that in addition to CX3CR1, synovial lining macrophages can be identified and isolated by the expression of VSIG4. Fig. S2 shows activation of lining macrophages by GO assessment at the onset of AIA and by CXCL1 protein in situ staining. Fig. S3 shows assessment of endothelial activation in the lining as well as reduced inflammation and pathology in either global or lining macrophage–specific IRF5KO. Table S1 lists the antibodies used in imaging and FACS experiments.

## Supplementary Material

Table S1 shows antibodies used in imaging and FACS experiments.

## Data Availability

The data supporting the findings of this study are available within the article and the main figures or its supplementary materials. Raw microscopy images demonstrated in the figures and used to generate plots are available upon request from the corresponding author (K. Zec). RNA sequencing data has been made publicly available under Gene Expression Omnibus accession number GSE229189.
