# Supplementary material for: Macrophages in the synovial lining niche initiate neutrophil recruitment and articular inflammation
Source: J Exp Med. 2023 Apr 28;220(8):e20220595. doi: 10.1084/jem.20220595 (PMC10148166; doi:10.1084/jem.20220595)
Supplement: Table S1 — shows antibodies used in imaging and FACS experiments. [file JEM_20220595_TableS1.docx]

| Antibody | Catalogue code | Manufacturer |
| --- | --- | --- |
| **Primary antibodies for confocal microscopy imaging** | | |
| GFP Rabbit IgG Polyclonal Antibody | A11122 | Life Technologies |
| Mouse VSIG4 Affinity Purified Polyclonal Ab | AF4674 | Bio-Techne (R&D Systems) |
| CD68 (FA-11), purified | MCA1957GA | Bio-Rad |
| Alexa Fluor® 647 anti-mouse CD68 (FA-11) | 137003 | BioLegend Inc |
| BV421 Rat Anti-Mouse CD68 | 566389 | BD Biosciences |
| Mouse CXCL1/GRO alpha/KC/CINC-1 Alexa Fluor 647 Antibody | IC4532R-100UG | Bio-Techne (R&D Systems) |
| Normal Rabbit IgG Alexa Fluor 647 Control (Cl 60024B) | IC1051R | Bio-Techne (R&D Systems) |
| Alexa Fluor® 647 anti-mouse CD31 (MEC13.3) | 102516 | BioLegend Inc |
| Alexa Fluor® 594 anti-mouse CD31 (MEC13.3) | 102520 | BioLegend Inc |
| CD31, purified (MEC 13.3) | 15898888 | Fisher Scientific UK Ltd |
| BV421 CD31 (MEC 13.3) | 562939 | BD Biosciences |
| Mouse CD177 MAb (1171A) | MAB8186-SP | Bio-Techne (R&D Systems) |
| Purified NA/LE Rat Anti-Mouse CD62E | 553748 | BD Biosciences |
| Anti-Lubricin/MSF antibody (Prg4) | ab28484-50ug | Abcam plc |
| **Secondary antibodies for confocal microscopy imaging** | | |
| Brilliant Violet 421™ Goat anti-rat IgG (minimal x-reactivity) | 405414 | BioLegend Inc |
| BV421 Goat Anti-Rat IgG | 565013 | BD Biosciences |
| Alexa Fluor 647 Goat Anti-Rat IgG (H+L)-0.5 mL | A21247 | Life Technologies Ltd |
| Alexa Fluor® 488 Goat anti-Rat IgG (H+L) Secondary Antibody | A11006 | Life Technologies Ltd |
| Alexa Fluor® 594 Goat Anti-Rat IgG (H+L) | ab150160-500ug | Abcam plc |
| BV421 Goat Anti-Rabbit IgG | 565014 | BD Biosciences |
| Alexa Fluor 647 Goat anti-Rabbit IgG (H+L), Novex SuperClonal Secondary Antibody | A27040 | Life Technologies Ltd |
| Alexa Fluor 488 Goat Anti-Rabbit IgG (H+L) | A11008 | Life Technologies |
| Alexa Fluor® 594 Goat Anti-Rabbit IgG (H+L) | ab150080-500ug | Abcam plc |
| Alexa Fluor 555 Goat Anti-Rabbit IgG (H+L) | A21428 | Life Technologies Ltd |
| Alexa Fluor Plus 647, Donkey anti-Rabbit IgG (H+L) Highly Cross-Adsorbed Secondary Antibody | A32795 | Life Technologies Ltd |
| Alexa Fluor 647 Donkey Anti-Goat IgG (H+L) Antibody | A21447 | Life Technologies Ltd |
| Brilliant Violet™ 421-AffiniPure Donkey Anti-Goat IgG (H+L) | 705-675-147-JIR | Stratech Scientific Ltd |
| Alexa Fluor Plus 594, Donkey anti-Goat IgG (H+L) Highly Cross-Adsorbed Secondary Antibody | A32758 | Life Technologies Ltd |
| Alexa Fluor 488 Donkey anti-Rabbit IgG (H+L) Highly Cross-Adsorbed Secondary Antibody | A-21206 | Life Technologies Ltd |
| **Antibodies used for FACS** | | |
| **APC VSIG4 Antibody (NLA14)** | 17-5752-82 | Life Technologies Ltd |
| **Brilliant Violet 650™ anti-mouse CD45** | 103151 | BioLegend Inc |
| **BV510 anti-human/mouse CD11b** | 101245 | BioLegend Inc |
| **PE-Cyanine7 F4/80 Antibody (BM8)** | 25-4801-82 | Life Technologies Ltd |
| **PerCP/Cy5.5 anti-mouse CD3e** | 100328 | BioLegend Inc |
| **PerCP/Cy5.5 anti-mouse CD19** | 115534 | BioLegend Inc |
| **PerCP/Cy5.5 anti-mouse NK-1.1** | 108728 | BioLegend Inc |
| **Brilliant Violet 711™ anti-mouse Ly-6G** | 127643 | BioLegend Inc |
| **Brilliant Violet 785™ anti-mouse Ly-6C** | 128041 | BioLegend Inc |
| **Recombinant Anti-IRF5 antibody** | ab181553 | Abcam plc |
| **Alexa Fluor 555 Goat Anti-Rabbit IgG (H+L)** | A21428 | Life Technologies Ltd |

**Table S1. Antibodies used in imaging and FACS experiments**
